# Mode I Fracture Toughness of Graphene Reinforced Nanocomposite Film on Al Substrate

**DOI:** 10.3390/nano11071743

**Published:** 2021-07-01

**Authors:** Shiuh-Chuan Her, Kai-Chun Zhang

**Affiliations:** Department Mechanical Engineering, Yuan Ze University, Chung-Li 320, Taiwan; s1025045@mail.yzu.edu.tw

**Keywords:** graphene nanoplatelet, nanocomposite film, fracture toughness, interfacial crack

## Abstract

Nanocomposites were prepared by adding graphene nanoplatelets (GNP) into epoxy with a variety of loadings. The thickness of GNPs used in this study was in a range of 1 nm to 10 nm. Nanocomposite film was deposited on the aluminum (Al) substrate via a spinning coating process. Tensile tests were carried out to determine the elastic modulus, ultimate strength and fracture strain of the nanocomposites. Theoretical prediction of the fracture toughness of the film/substrate composite structure with an interfacial crack under mode I loading was derived utilizing linear elastic fracture mechanics theory. Four-point bending tests were performed to evaluate the mode I fracture toughness. It was observed that the performance of the nanocomposite, such as elastic modulus, ultimate strength, and fracture toughness, were significantly enhanced by the incorporation of GNPs and increased with the increase in GNP concentration. The elastic modulus and mode I fracture toughness of the epoxy reinforced with 1 wt.% of GNPs were increased by 42.2% and 32.6%, respectively, in comparison with pure epoxy. Dispersion of GNPs in the epoxy matrix was examined by scanning electron microscope (SEM). It can be seen that GNPs were uniformly dispersed in the epoxy matrix, resulting in the considerable improvements of the ultimate strength and fracture toughness of the nanocomposite.

## 1. Introduction

Since the discovery of graphene in 2004 by Novoselov and Geim [1,2], graphenes have received tremendous attention from both academia and industry due to their excellent electrical, mechanical, and thermal properties. Graphene consists of carbon atoms laid in a single layer with a thickness of one atom, bonded by $sp^{2}$ to form a hexagonal structure, leading to extraordinary in-plane functional and mechanical properties. It has been considered as a promising nanofiller for reinforcement. Graphene exhibits better performance in comparison with other fillers such as carbon black, carbon nanotube and nano-silica owing to its outstanding mechanical strength and electrical conductivity. Rafiee et al. [3] investigated mechanical properties of epoxy based nanocomposites with multi-walled carbon nanotube (MWCNT), single-walled carbon nanotube (SWCNT) and GNP additives. They reported that the tensile strength of the nanocomposites reinforced with 0.1 wt.% of MWCNT, SWCNT, and GNP was improved by 14%, 11%, and 40%, respectively, in comparison with pristine epoxy. Rafiee et al. [4] studied the buckling of nanofillers reinforced nanocomposite beam. They found that incorporation of 0.1 wt.% MWCNT, SWCNT, and GNP into the epoxy resin, the critical buckling load was increased by 6.2%, 15%, and 52%, respectively, in comparison with neat epoxy. Chandrasekaran et al. [5] reported the increases in fracture toughness for MWCNT/epoxy and GNP/epoxy nanocomposites with 0.5 wt.% of nanofillers were 8% and 25%, respectively, while compared with pure epoxy. Polymer based nanocomposites have been successfully used in many industries especially in the fields of aerospace, automobile, electronic, and wind turbine. Graphene exhibits a larger surface to volume ratio in comparison with CNT, making it more effective for enhancing the electrical, thermal, and mechanical properties of the polymer based matrices [6]. Moreover, the cost of GNP is less expensive than that of CNT, since GNP can be easily fabricated from a graphite in a large quantity. Polymer/graphene nanocomposites have attracted a great attention in scientific community due to significant improvement of mechanical property at very low fractions [7,8,9]. Liang et al. [10] reported an increase of 76% in ultimate strength accompanied by an increase of 62% in elastic modulus for poly (vinyl alcohol) epoxy composite incorporation with 0.7 wt.% of graphene. Zhao et al. [9] illustrated an increase of 150% in ultimate strength with 1.8 wt.% of graphene in poly (vinyl alcohol) nanocomposites.

Epoxy has been extensively used in many engineering applications including wear protection, adhesives, encapsulation for electronic devices, and as a polymer matrix for nanofillers reinforced nanocomposite [11]. Epoxy based nanocomposites reinforcing with GNPs have been experimentally studied by many researchers [5,12,13]. The results depict significant improvement in fatigue, mechanical, and thermal properties. It was observed that the degree of dispersion of GNPs in the epoxy resin is a critical issue in the determination of nanocomposite properties. GNP has an intrinsic tendency of agglomeration due to van der Waals force in conjunction with a large surface area. Moreover, π-π bonding interactions among the carbon atoms enhance the stacking of GNPs. The agglomeration of GNPs in the polymer matrix due to the poor dispersion not only considerably reduces their reinforced efficiency but also causes slippage between the neighboring graphene, resulting in a decrease in load transfer. In recent years, a variety of surface modification technologies of graphene have been proposed to enhance the dispersion and interfacial interaction of graphene in polymer matrix. Fang et al. [14] modified GNPs with aromatic amines functional group which were covalently bonded on graphene to prepare graphene/epoxy nanocomposites, resulting in remarkable enhancements in flexural strength and modulus by 54% and 60%, respectively. Teng et al. [7] used pyrene molecules non-covalent stacking on the surface of graphene through the π-π bonds, leading to a significant improvement of the thermal conductivity for the epoxy matrix. Epoxy exhibits superior mechanical properties, corrosion, and chemical resistance owing to the high crosslink between the epoxy and curing agent. The high degree of crosslink makes epoxy brittle and easy to break. A variety of reinforcements such as liquid rubbers [15], hydroxyl-terminated poly (ether ether ketone) [16], silica [17], and clay [18] have been introduced to enhance the fracture toughness of epoxy matrix. Although the incorporation of rubbers and thermoplastic polymers significantly improved the fracture toughness of the epoxy matrix, the high loadings of these reinforcements can reduce the other mechanical and thermal properties, such as stiffness, strength, and glass transition temperature [11]. To overcome this issue, high stiffness nanofillers such as CNTs [19] and GNPs [20] have been employed to toughen epoxy.

Most of existing literatures focused on the improvements of Young’s modulus and tensile strength for GNP reinforced nanocomposites. Fracture toughness is rarely studied, in particular for the interfacial fracture toughness of a nanocomposite film/substrate composite structure. Epoxy deposited on a substrate has a great potential application in adhesive bonding and protective coating. Debonding between the film and substrate is critical and required intensive study to meet the structural safety requirement. The novelty of present work is that a theoretical model in combination with experimental test were proposed to reveal the reinforcement effect of GNP on the interfacial fracture toughness of the nanocomposite film/substrate composite structure. Adhesively bonded joint between two components is a practical application of film/substrate composite structure which can be subjected a variety of loadings including tension, compression, and bending in service. In this work, the graphene/epoxy nanocomposite was prepared via a surfactant assisted process. Triton X-100 was employed to modify the GNPs to increase the compatibility and wettability within epoxy resin. Geng et al. [21] has demonstrated the effectiveness of Triton X-100 for the dispersion of CNTs in epoxy resin. Moreover, the dispersion of GNPs in the epoxy resin was conducted using a tip sonication. Nanocomposite film was deposited on the Al substrate via a spinning coating process. Theoretical prediction of the fracture toughness of the film/substrate composite structure with an interfacial crack under mode I loading was derived utilizing linear elastic fracture mechanics theory. The mode I fracture toughness was experimentally determined using a four-point bending test. The influence of GNP concentration on the fracture toughness, elastic modulus and ultimate strength of the nanocomposite was investigated.

## 2. Materials and Methods

### 2.1. Nanocomposite Preparation

**Figure 1:**
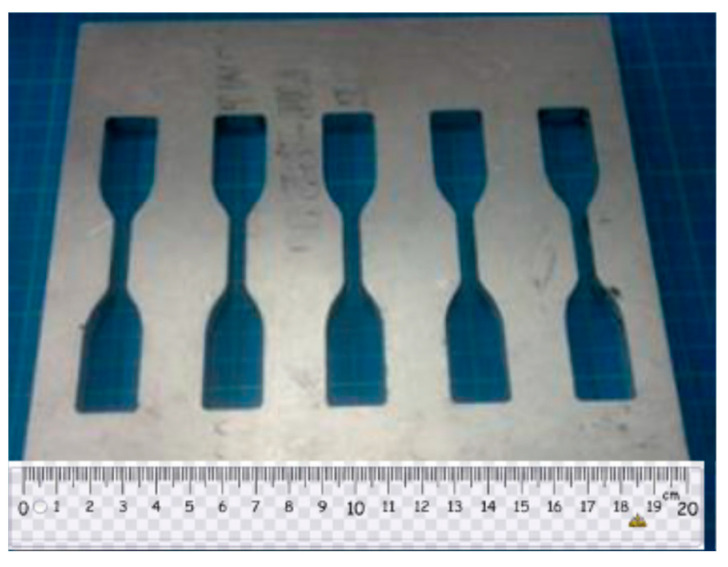
Aluminum mold for tensile test specimen.

**Figure 2:**
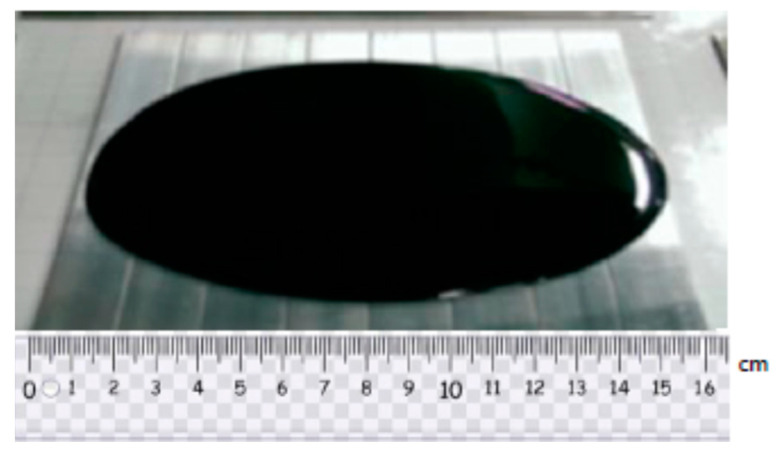
Nanocomposite deposited on Al substrate.

The graphene nanoplatelets (GNP) were purchased from Uchess Co. (New Taipei City, Taiwan). The thickness of GNP was varying from 1 nm to 10 nm, while the lateral dimension was in a range of 0.5 μm to 20 μm. The epoxy used in this work was Mungo 4200A part A and hardener 4200B part B provided by Golden Root Co., Ltd. (Taipei City, Taiwan). The mixed ratio between the epoxy resin and hardener was 2:1 in weight according to the recommendation of the manufacturer. To decrease the viscosity of the liquid epoxy, ethanol was added into the epoxy. After incorporation of GNPs into the liquid epoxy, the dispersion was performed by a sonicator (Q700, Qsonica L.L.C., Newtown, CT, USA) through a horn sonication process. The sonication prob was immersed in the mixture and worked at a pulse mode with 10 s on and 20 s off for 20 min. Consequently, the hardener was incorporated into the mixture, and manually mixed for 20 min. After that, the GNP/epoxy mixture was placed in a vacuum chamber at a constant temperature of 25 °C for 60 min to degas the trapped air due to the stirring. The degassed nanocomposite was poured into an aluminum mold, as illustrated in Figure 1, to prepare the tensile testing specimen. In addition, the degassed nanocomposite was deposited on the Al substrate as depicted in Figure 2 which was held on a spinning coating machine (RMT-SC 150SS, Reliable-Mate Technology Co. Ltd., Shin-Chu City, Taiwan). The film thickness of the nanocomposite deposited on the Al substrate can be modulated by varying the rotating speed of the spinning coating machine. The nanocomposite was cured in a thermal chamber at a constant temperature of 40 °C for 24 h. In this work, the film thickness deposited on the Al substrate was kept at a constant of 0.3 mm. Nanocomposites with four different GNP concentrations varying from 0.3 to 1 wt.% were prepared to investigate the effect of GNP content on the mode I fracture toughness and tensile mechanical properties.

### 2.2. Fracture Surface Morphology

**Figure 3:**
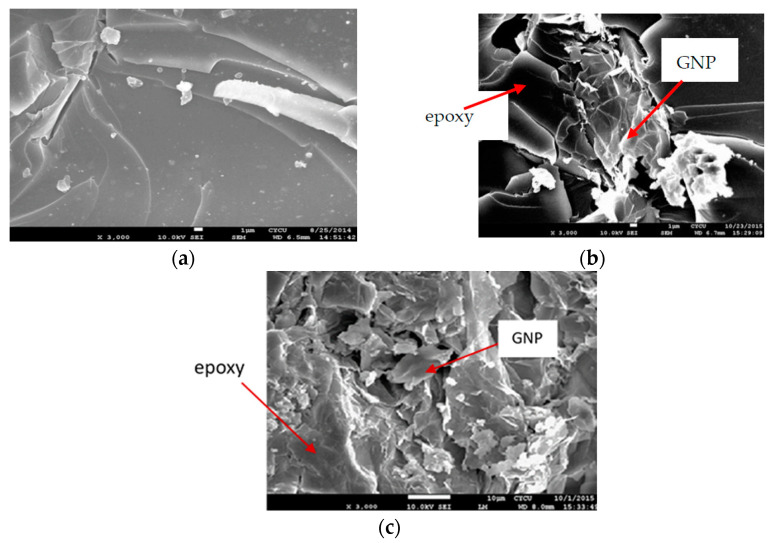
SEM images of the fracture surfaces (**a**) neat epoxy; (**b**) nanocomposite incorporated with 0.5 wt.% GNP; (**c**) nanocomposite incorporated with 1.0 wt.% GNP.

It is well known that carbon based nanofillers such as carbon nanotube (CNT) and graphene nanoplatelet (GNP) have a tendency of agglomeration owing to van der Waals force and π-π bond. Dispersion of GNPs in the matrix is a critical and challenging issue. The performance of the nanocomposite is heavily relied on the dispersion of nanofillers in the matrix. The dispersion of GNP in the epoxy matrix can be investigated through the morphology of the fracture surface. In this work, the morphology of the fracture surface of the nanocomposite was characterized via SEM (JSM 7600F, Jeol Co.,Tokyo, Japan). The specimen was coated with platinum and operated at an accelerated voltage of 10 kV. SEM images of the fracture surfaces for the neat epoxy, nanocomposites reinforced with 0.5 wt.% and 1.0 wt.% GNPs are illustrated in Figure 3a–c, respectively. It can be seen that the fracture surface for the neat epoxy exhibits a smooth morphology in comparison with GNP reinforced nanocomposites. This demonstrates that neat epoxy has a low resistance to the crack propagation. In addition, uniform dispersion of GNPs in the epoxy matrix can be conformed from the SEM images of the fracture surface. Thus, tensile mechanical properties of the nanocomposite can be enhanced by incorporation of GNPs into the epoxy matrix. Conversely, poor dispersion due to the GNP agglomeration leads to the formation of voids or defects, may reduce their reinforcement effect on the mechanical properties.

### 2.3. Mode I Fracture Toughness

**Figure 4:**
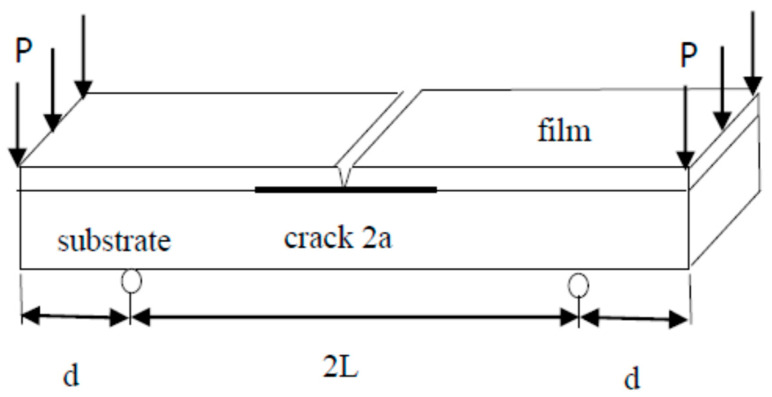
Film/substrate composite beam subjected to four-point bending.

**Figure 5:**
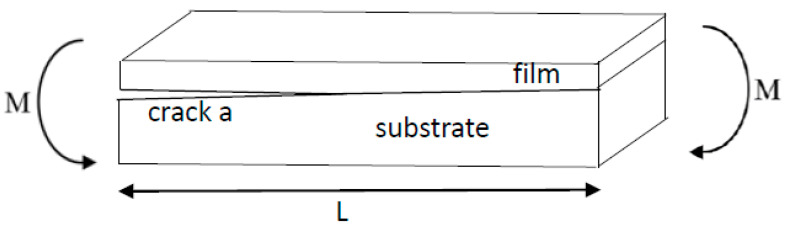
Pure bending moment exerted on the composite beam.

The mode I interfacial fracture toughness of film/substrate composite structure with a crack along the interface was derived based on the linear elastic fracture mechanics. Figure 4 illustrates the theoretical model of this study. To prepare an interfacial crack of the film/substrate composite structure, silicone oil was deposited on the central area of the Al substrate before the nanocomposite was poured onto the Al substrate. After the curing of nanocomposite, debonding between the nanocomposite film and Al substrate occurred in the central area where the silicone oil was deposited results in an interfacial crack. A notch was introduced to the middle of nanocomposite film so that a four-point bending loading exerted on the composite beam with interfacial crack can induce opening fracture mode. The film/substrate composite beam was under a four-point bending loading. The interfacial crack region which is located in the middle of the composite structure is subjected to a constant moment, leading to a mode I opening mode. The strain energy release rate of the composite beam exhibits in a steady state while the crack length is much longer than that of the thickness of the film. Utilizing the symmetric condition, the right half of the composite structure was subjected to a bending moment of *M* = *Pd* as depicted in Figure 5. As the crack grows from *a* to *a* + *δa*, the variation of the strain energy stored in the composite structure after the crack propagation can be calculated as follow.

(1) $$\delta W=\int_{0}^{\delta a}\frac{M^{2}}{2\bar{E}\bar{I}}dx-\int_{0}^{\delta a}\frac{M^{2}}{2E_{s}I_{s}}dx$$

(2) $$\bar{E}\;\bar{I}=\frac{b}{12}\left[E_{f}h_{f}^{3}+E_{s}h_{s}^{3}+3E_{f}E_{s}h_{f}h_{s}\frac{{\left(h_{f}+h_{s}\right)}^{2}}{E_{f}h_{f}+E_{s}h_{s}}\right]$$

where $E_{s}\;\mathrm{and}\;E_{f}$ denote the elastic moduli of the substrate and nanocomposite film, respectively; $h_{s}\;\mathrm{and}\;h_{f}$ represent the thicknesses of the substrate and nanocomposite film, respectively; *b* is the width of the composite beam; $\bar{E}\;\bar{I}$ is the flexural rigidity of the composite beam.

Strain energy release rate considered as the energy available for an increment of crack propagation is defined as follow.

(3) $$G_{I}=\underset{\delta A\to 0}{\lim}\left|\frac{\delta W}{\delta A}\right|=\underset{\delta a\to 0}{\lim}\left|\frac{\delta W}{b\delta a}\right|$$

Substituting Equation (1) into Equation (3) leads to the mode I strain energy release rate as follow.

(4) $$G_{I}=\frac{6M^{2}}{b^{2}}\left[\frac{1}{E_{s}h_{s}^{3}}-\frac{E_{f}h_{f}+E_{s}h_{s}}{\left(E_{f}h_{f}^{3}+E_{s}h_{s}^{3}\right)\left(E_{f}h_{f}+E_{s}h_{s}\right)+3E_{f}E_{s}h_{f}h_{s}{\left(h_{f}+h_{s}\right)}^{2}}\right]$$

## 3. Results and Discussions

### 3.1. Tensile Mechanical Properties

**Figure 6:**
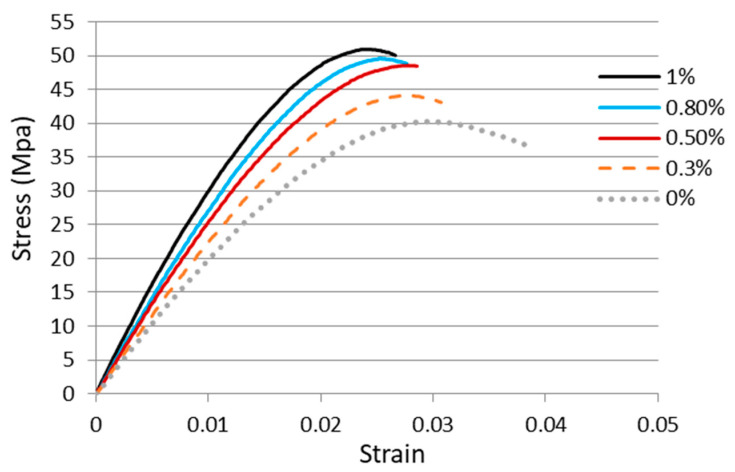
Stress vs. strain curves of the nanocomposites reinforced with various GNP contents.

**Figure 7:**
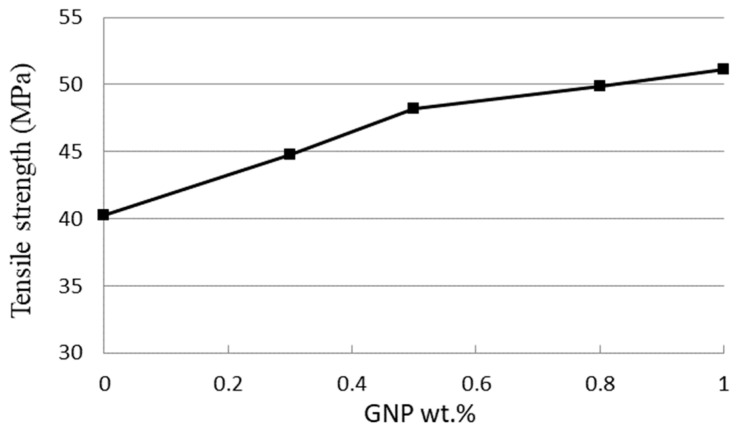
Tensile strength of nanocomposite reinforced with various GNP contents.

**Figure 8:**
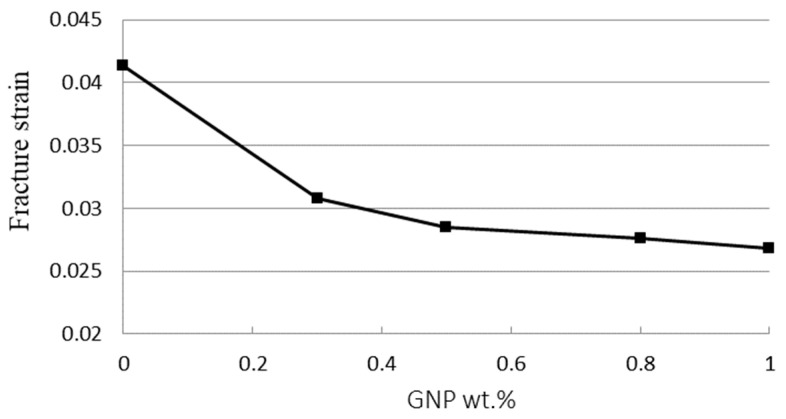
Fracture strain of nanocomposite reinforced with various GNP contents.

Tensile tests were employed to determine the mechanical properties including the elastic modulus, fracture strain, and tensile strength of the GNPs reinforced nanocomposites. In this work, experimental tests were conducted according to the ASTM standard D638 with a constant loading rate of 5 mm/min at room temperature by a tensile testing machine (H10KS, Hounsfield Test Equipment Ltd., Surrey, UK). Figure 6 shows the stress vs. strain curves of the nanocomposites with four different GNP concentrations in a range of 0.3 wt.% to 1 wt.% from the tensile tests. In addition, the stress vs. strain curve of the neat epoxy was also presented for the comparison. Utilizing the stress vs. strain curve, the elastic modulus of the nanocomposite was obtained from the slope of the linear elastic region, while the yield strength can be extracted using the 0.2% offset. Tensile mechanical properties of the nanocomposites incorporated with various contents of GNPs are listed in Table 1. It appears that the tensile properties such as the ultimate strength, yield strength, and elastic modulus are increased with the increasing of GNPs as illustrated in Figure 7, while the fracture strain exhibits in an opposite trend as shown in Figure 8. The tensile testing results demonstrate that the strength properties of the nanocomposite are enhanced by the incorporation of GNPs at the expense of ductility, i.e., low strain to fracture. The elastic modulus, yield strength, and ultimate strength of the nanocomposite reinforced with 1.0 wt.% of GNPs are improved by 42.2%, 27.2%, and 26.9%, respectively, in comparison with neat epoxy, while the fracture strain is decreased by 35.3%. The performance of nanocomposite is significantly affected by its individual components (nanofillers and polymer matrix). Lee et al. [22] reported a tensile strength of 130 GPa for GNP. The reinforced effect of GNPs in epoxy matrix is a complex issue. It involves several factors such as load transfer, stress concentration, and agglomeration. Among them, load transfer from GNP to epoxy matrix is a key issue to improve the elastic modulus and ultimate strength. Tang et al. [12] investigated the interface between the graphene and epoxy matrix using TEM. They found that GNP agglomeration due to a poor dispersion induced gap and debonding between the graphene and epoxy matrix, leading to stress concentration and reducing load transfer. The poorly and highly dispersed RGO (reduced graphite oxide) at 0.2 wt.% content increased 24% and 52% in stress intensity factor, respectively. It is well known that a strong interfacial interaction between the GNP and epoxy matrix play an important role to improve the load transfer. On the contrary, stress concentration and agglomeration reduce the reinforcement. Poor dispersion causes the formation of micro voids, leading to the stress concentration in the epoxy matrix. Stacking behavior of GNPs owing to a large surface area in conjunction with the van der Waals force make GNPs tend to agglomerate. Slippage of the overlapped GNPs can be occurred while the tensile load is applied. As a result of the load transfer from the GNP to the epoxy matrix is reduced. Thus, the improvements in the elastic modulus and ultimate strength of the GNP reinforced nanocomposite are attributed to the strong interfacial interaction and uniform dispersion of GNPs in the epoxy resin.

### 3.2. Fracture Toughness

**Figure 9:**
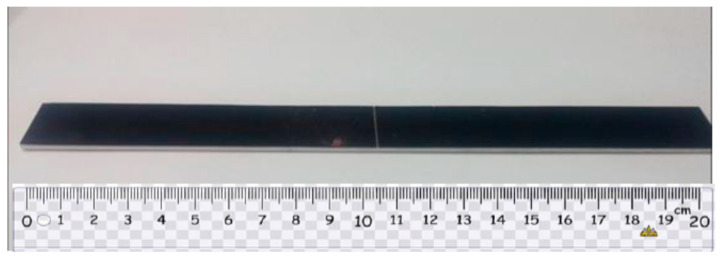
As prepared composite beam with an interfacial crack.

**Figure 10:**
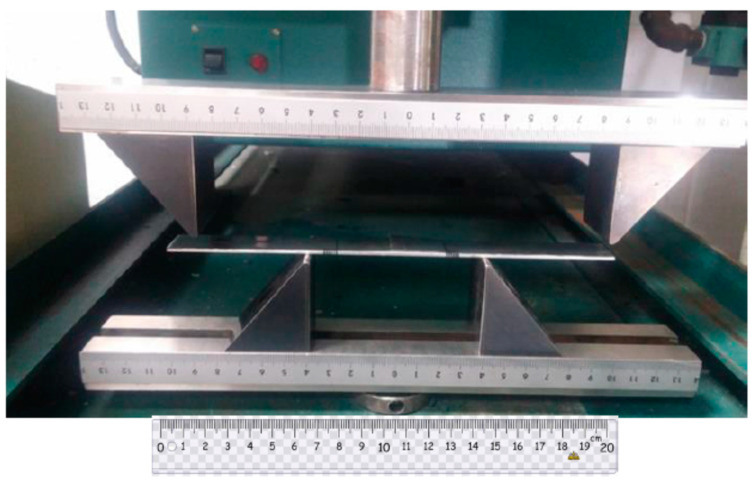
Four-point bending test.

**Figure 11:**
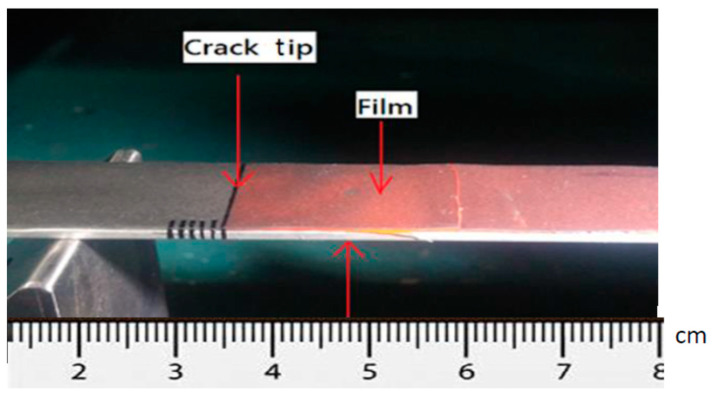
Red ink dropped into the unbonded region of the composite beam.

**Figure 12:**
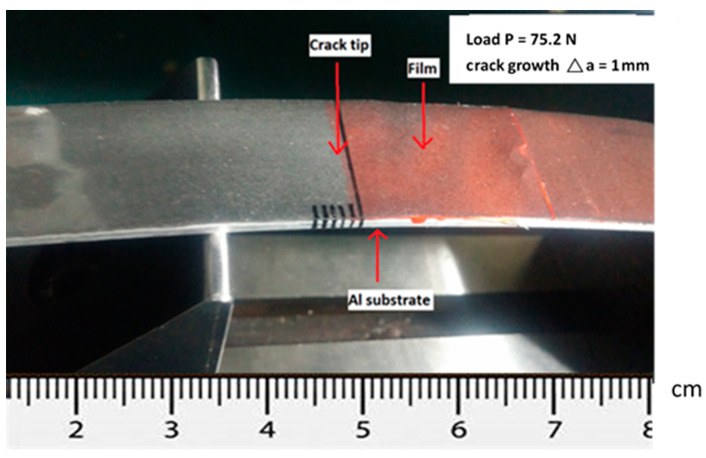
Interfacial crack propagation initiated at the critical load.

**Figure 13:**
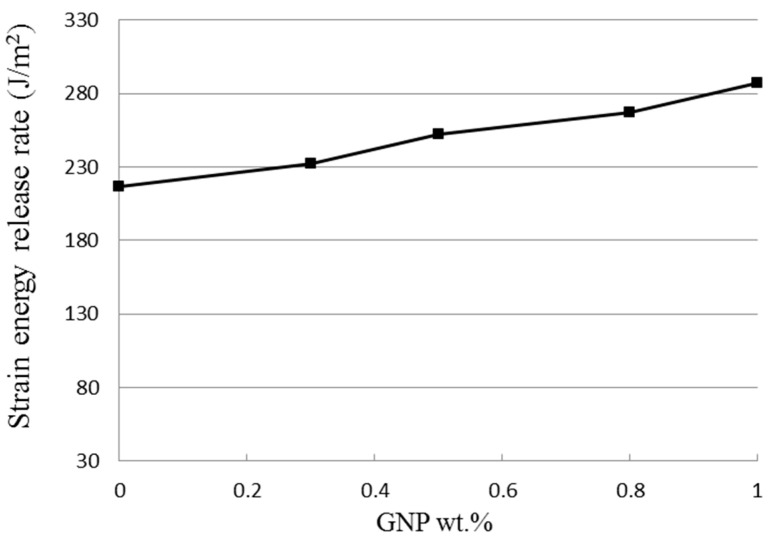
Strain energy release rate of nanocomposite varied with GNP contents.

The theoretical derivation of the mode I fracture toughness for the film/substrate composite beam with an interfacial crack was presented in Section 2.3 as shown in Equation (4). In this work, experimental tests were conducted to determine the mode I fracture toughness based on the four-point bending test. The geometrical dimensions of the test specimen were 200 mm in length and 19 mm in width. The thicknesses for the Al substrate and nanocomposite film were 2 mm and 0.3 mm, respectively. The length of the interfacial crack was 50 mm. The as prepared composite beam is illustrated in Figure 9, consisting of a nanocomposite film on the Al substrate with an interfacial crack. The film/substrate composite beam was under a four-point bending test as depicted in Figure 10. The test specimen was placed on a four-point bending fixture. There is a ruler on the top and bottom of the fixture as shown in Figure 10. Thus, the test specimen can be adjusted and placed on the correct position with the aid of rulers. The distance between the two outer loadings was 180 mm, while the distance between the two simply supports was 80 mm. A bending moment of *M* = *P* × *d* was applied on the middle region of the composite beam where *P* is the applied load and *d* = *50* mm. The load was slowly increased to reach a critical load $P_{cr}$ which initiates the propagation of the interfacial crack. Since the GNP/epoxy nanocomposite exhibits a black color even at a very low concentration of GNP, it is difficult to identify the crack tip. To overcome this issue, red ink was dropped into the unbonded region where the interfacial crack was occurred as shown in Figure 11. The crack and uncrack regions can be distinguished by the red and black colors, respectively. The contrast in colors make the observation of crack propagation easier. The interfacial crack propagation was initiated at the critical load $P_{cr}$ as shown in Figure 12. The mode I strain energy release rate $G_{Ic}$ is readily to be evaluated by substituting the critical load $P_{cr}$ into Equation (4). Three test specimens were prepared and performed the four-point bending tests for each GNP concentration. The average of the three test results was presented in this work. The mode I fracture toughness of nanocomposite film reinforced with GNPs ranging from 0 wt.% to 1 wt.% are presented in Table 2. It can be observed that the mode I interfacial fracture toughness of film/substrate composite structure is increased with the increasing of GNP concentration in nanocomposite film as depicted in Figure 13. The mode I fracture toughness of the nanocomposite film reinforced with 1.0 wt.% of GNP is enhanced by 32.6% while compared with pure epoxy. Fracture toughness is considered as a measurement of energy required to initiate crack propagation which is an important property relative to the structural safety. The toughening effect of GNPs in the epoxy resin may be attributed to: (i) excellent strength of GNP; (ii) good dispersion in epoxy resin; (iii) strong interfacial interaction between the GNPs and epoxy. Two-dimensional geometry of GNP exhibits a large aspect ratio and surface area. This makes GNP more accessible to share loads and prevent crack propagation in comparison with other nanofillers, such as CNT and carbon black. The morphology of the fracture surface of neat epoxy is very smooth, as shown in Figure 8a. It demonstrates brittle fracture characteristics with rapid crack propagation after the crack initiation. On the contrary, the fracture surface of nanocomposite reinforced with GNPs appears in a coarser morphology as shown in Figure 3b,c. It is noted that GNPs provide obstacles to the crack propagation. Crack path in the epoxy matrix can be affected by the incorporation of GNPs. When crack propagation proceeds to meet GNPs, crack is deflected and continue to propagate along three possible paths: (1) crack wraps around the GNP; (2) crack splits into two cracks to detour GNP; (3) crack penetrates in between the layers to split the agglomerate GNPs in two. These damage mechanisms were also reported by Mefford et al. [23] and Chandrasekaran et al. [24]. Thus, these crack propagation processes cause the crack to take more tortuous paths, leading to a rough surface and consuming more fracture energy for free surface energy according to Griffith model. Thus, incorporation of GNPs into epoxy matrix can effectively prevent the crack propagation and consume more energy, leading to a remarkable enhancement of the fracture toughness.

## 4. Conclusions

In this work, GNP reinforced nanocomposite was prepared and successfully deposited on an Al substrate using horn sonication in combination with spinning coating technologies. The thickness of GNPs was ranging from 1 nm to 10 nm. Theoretical prediction of mode I fracture toughness of nanocomposite film on an Al substrate was derived utilizing linear elastic fracture mechanics theory. Tensile and bending tests were carried out to evaluate the tensile properties and mode I fracture toughness of the nanocomposite, respectively. It was observed that the tensile strength and fracture toughness of the nanocomposite incorporated with 1.0 wt.% of GNPs were increased by 26.9% and 32.6%, respectively, in comparison with pure epoxy. The reinforced effect of GNP may be attributed to uniform dispersion in conjunction with good interfacial interaction between the GNP and epoxy matrix for enhancing the load transfer and preventing crack propagation. SEM images of the fracture surfaces demonstrated a good dispersion of GNPs and also confirmed the toughen mechanism for the improvement of the fracture toughness. Adhesively bonded joint between two components is a practical application of film/substrate composite structure which can be subjected a variety of loadings including tension, compression, and bending in service. Present approach provides a theoretical model and experimental test to evaluate the fracture behavior of adhesively bonded joints.

## Figures and Tables

**Table 1:** Tensile properties of the nanocomposites incorporated with a variety of GNP concentrations.

Tensile Properties	Neat Epoxy	Nanocomposites			
		0.3 wt.% GNP	0.5 wt.% GNP	0.8 wt.% GNP	1.0 wt.% GNP
Elastic modulus (GPa)	1.99	2.33	2.49	2.69	2.83
Yield strength (MPa)	31.56	35.82	37.04	38.86	40.13
Tensile strength (MPa)	40.33	44.75	48.24	49.87	51.16
Fracture strain	0.0414	0.0308	0.0285	0.0276	0.0268

**Table 2:** Mode I fracture toughness of the nanocomposite film reinforced with various GNP concentrations.

GNP wt.%	Specimen 1		Specimen 2		Specimen 3		Average Strain Energy Release Rate $G_{I}$ (J/m^2^)
	Critical Load $P_{cr}$ (N)	Strain Energy Release Rate	Critical Load $P_{cr}$ (N)	Strain Energy Release Rate	Critical Load $P_{cr}$ (N)	Strain Energy Release Rate	
		$G_{I}$ (J/m^2^)		$G_{I}$ (J/m^2^)		$G_{I}$ (J/m^2^)	
0%	68.6	216.14	72.8	218.12	70.6	216.66	216.97 ± 0.83
0.3%	75.2	233.52	74.4	231.25	77.5	232.65	232.47 ± 1
0.5%	83.2	251.63	86.5	251.21	86.1	253.39	252.07 ± 0.86
0.8%	89.5	266.21	89.2	265.83	92.5	266.47	267.17 ± 1.34
1%	103.1	285.65	99.5	288.56	101.2	285.52	286.58 ± 1.98

## Data Availability

Data available on request.
